# Coupled cycling and regulation of metazoan morphogenesis

**DOI:** 10.1186/s13008-020-0059-3

**Published:** 2020-01-27

**Authors:** Saba Rezaei-Lotfi, Ramin M. Farahani

**Affiliations:** 1 IDR/Westmead Institute for Medical Research, Westmead, Australia; 2 Faculty of Medicine and Health, University of Sydney, Sydney, NSW 2006 Australia

**Keywords:** Cell cycle, β-Catenin, Synchronisation, Self-organisation

## Abstract

Metazoan animals are characterized by restricted phenotypic heterogeneity (i.e. morphological disparity) of organisms within various species, a feature that contrasts sharply with intra-species morphological diversity observed in the plant kingdom. Robust emergence of morphogenic blueprint in metazoan animals reflects restricted autonomy of individual cells in adoption of fate outcomes such as differentiation. Fates of individual cells are linked to and influenced by fates of neighboring cells at the population level. Such coupling is a common property of all self-organising systems and propels emergence of order from simple interactions between individual cells without supervision by external directing forces. As a consequence of coupling, expected functional relationship between the constituent cells of an organ system is robustly established concurrent with multiple rounds of cell division during morphogenesis. Notably, the molecular regulation of multicellular coupling during morphogenic self-organisation remains largely unexplored. Here, we review the existing literature on multicellular self-organisation with particular emphasis on recent discovery that β-catenin is the key coupling factor that programs emergence of multi-cellular self-organisation by regulating synchronised cycling of individual cells.

## Background

Developmental morphogenesis describes cellular and molecular events that instruct the final shape and size of metazoan tissues/organs together with functional specialization of the constituent cells [1]. During morphogenesis, sequential rounds of mitosis subsequent to the formation of a zygote generate a homogenous population of totipotent cells that gradually and iteratively commit to their individual fates via differentiation [2]. The molecular basis of the differentiation program has been studied extensively and several models have been proposed in an attempt to explain the gradual commitment to differentiation of individual proliferating cells [2, 3]. It is generally believed that the morphogenic blueprint of an organism is genetically encoded and phenotypically interpreted at the level of individual cells. That is to say individual cells access their DNA and selectively retrieve the genetic information to drive differentiation in a step-wise manner during ontogeny. The basic tenet of such a centralized (i.e. cell-autonomous) model of morphogenesis is the assumption that the DNA content of individual cells in an organism is nearly identical. The emerging evidence, however, has started to portray a different image whereby significant genomic variability can exist between individual cells of an organism [4–6]. In the face of genomic variability, robust emergence of a developmental blueprint suggests that interpretation of morphogenic programs is accomplished in a decentralized manner and at a platform higher than the level of individual cells. We recently investigated the biological platform that governs decentralized spatial, temporal and functional organisation of metazoan cells [7]. Findings revealed that multicellular organisation during neurogenesis is driven by an unexpected evolutionary adaptation of metazoan cell cycle. Unlike autonomous cycling of unicellular organisms, metazoan cell cycle is coupled to those of neighboring cells [7]. In a coupled cycling mode, intercellular contacts relay extrinsic cues to override the intrinsic cycling rhythm of an individual cell. In consequence, a form of collective behavior emerges that is characterized by synchronised cycling of individual cells. As cell cycle dynamics instructs downstream decisions such as differentiation outcome [8] and spatial navigation [9], the synchronised cycling mode can minimize phenotypic heterogeneity that results from genomic variability of individual cells in an organism. Herein, we address the latter decentralized mode of morphogenesis, referred to as cellular self-organisation.

## Self-organisation: a decentralized decision-making platform

Self-organisation describes emergence of order in a system without supervision by external directing forces [10]. Self-organisation is a decentralized decision-making strategy whereby local interactions between components of a system guide gradual emergence of global (i.e. system-level) order. Many micro- and macro-scale phenomena are orchestrated by self-organising principles [11]. In schooling fish, for example, adoption of the predominant movement direction by individual fish leads to eventual emergence of synchronised swimming. From a teleological perspective, reliance on self-organisation renders an evolving system more robust and less context-dependent. Such robustness results from the fact that local perturbations during the evolution of a self-organising system are subsequently corrected by feedback from other elements of the system. In other words, bidirectional exchange of information between components of a self-organising system leads to suppression of the autonomous decision making capacity of the individual components. The advantage of decentralized decision-making can be clearly illustrated by comparing the impact of applying an external force on multiple oscillating pendulums in coupled and uncoupled modes. In the uncoupled state, application of external load would perturb the impacted pendulum. In the coupled state, however, the impact of external force would be dissipated to all oscillating pendulums and would eventually be neutralized. Similarly, self-organisation principles that govern emergence of order (anatomy and function) during developmental morphogenesis [12] ought to rely upon a coupling agent that could transmit information between cycling cells. Recent work in our laboratory provided evidence that β-catenin demonstrates such coupling capacity.

## β-Catenin and coupling of cell cycle

β-Catenin is a metazoan cytoskeletal protein that can also function as a transcriptional factor upon translocation to the nucleus [13]. In order to stabilize intercellular contacts, β-catenin partners with α-catenin and connects cadherins to the actin cytoskeleton [14, 15]. The stability of catenin-cadherin assembly is regulated by integration of input from diverse signaling cascades [16, 17]. For example, phosphorylation of β-catenin Tyr-142 by Fyn tyrosine kinase leads to disruption of its association with α-catenin and subsequent release of the unbound protein into the cytoplasm. Upon dissociation from junctional complexes, free cytoplasmic β-catenin is tightly regulated by a destruction complex that recruits the protein and degrades it subsequent to phosphorylation by Casein kinase-I and Gsk-3β [18]. As such, free β-catenin is rapidly degraded (half-life ≈ 1 h) [19] or is alternatively shuttled into the nucleus where it functions as a transcription factor for select genomic loci [20]. Two major drivers of cell cycle, cyclin-D1 [21] and C-myc [22], are amongst the genes that are trans-activated by β-catenin. Cyclin-D1 facilitates progression though G1 phase [23] upon association with cdk4,6, an event that leads to phosphorylation of Retinoblastoma protein and activation of E2F family of transcription factors [24]. Myc, on the other hand, stimulates transcription of genes involved in growth and proliferation [25]. Myc enhances RNAPI-mediated transcription of 18S, 5.8S, and 28S rRNAs [26] and RNAPII-mediated transcription of ribosomal proteins [27]. The net outcome of the transcriptional activity of Myc is enhanced ribosome biogenesis and global protein synthesis that primes the cell for subsequent S phase [28]. It therefore is not surprising that amplified activity of β-catenin accelerates progression through cell cycle by installing a truncated G1 phase [29]. Contrarily, recruitment of β-catenin to adherens junctions [14, 15] delays the entry to S phase of G1 dwelling cells in a cell density-dependent manner [7]. The described activity of β-catenin in coupling the individual cell cycles across a proliferating population is the basis for synchronised cycling of metazoan cells.

## Molecular basis of coupled cycling

**Fig. 1:**
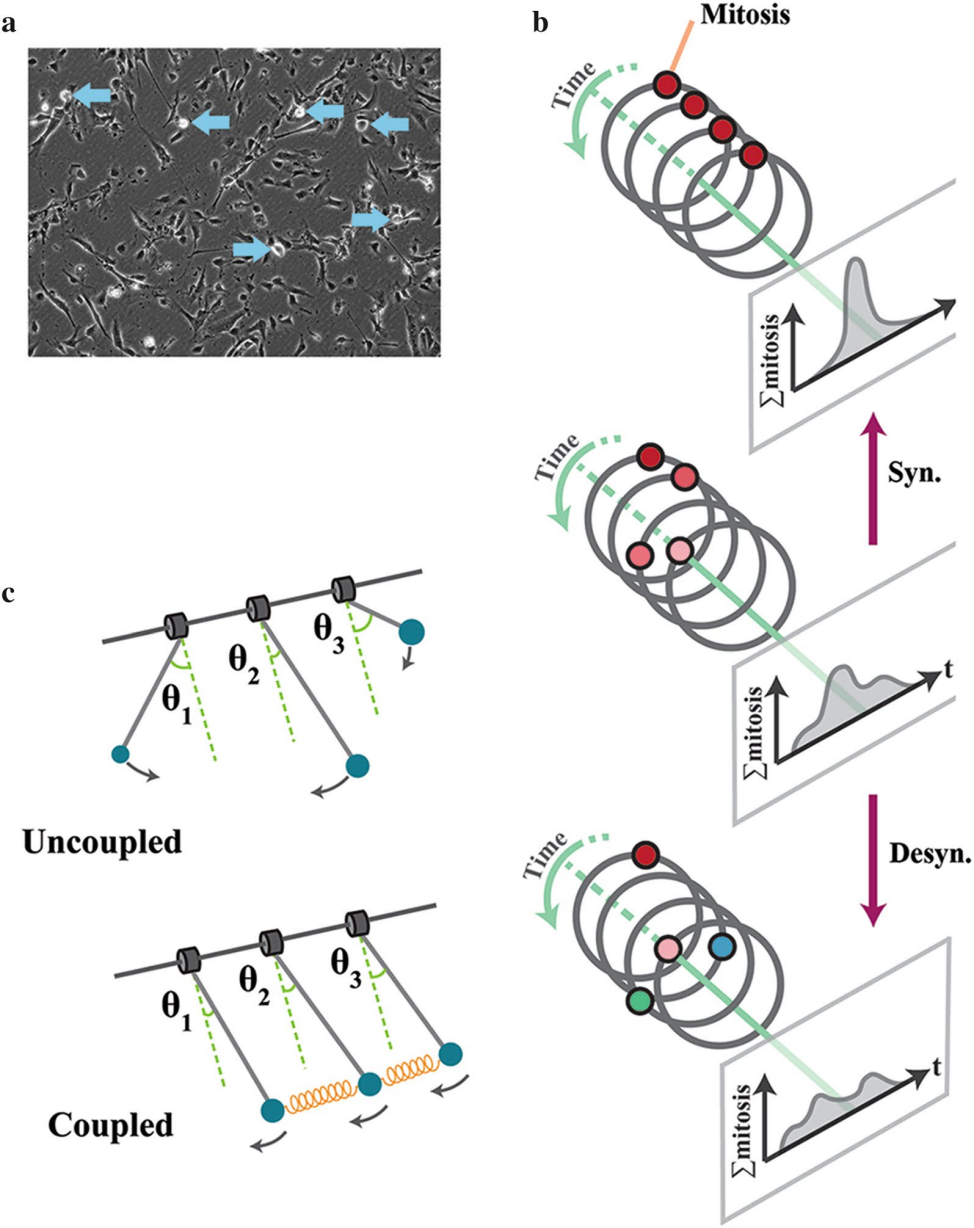
Coupled oscillation as the basis for synchronisation. **a** Live imaging shows synchronised mitotic rounding in cultured neural progenitor cells with partial synchronisation of cell cycle. **b** Oscillating pendulums move in a synchronised manner subsequent to installation of a weak coupler (orange spring). **c** Similar to coupled pendulums, coupled cycling cells enter mitosis in a synchronised manner

A key signature of human neural progenitor cells [30] cultured in 2D monolayers is partial synchronicity of the entry into mitosis at population level [7]. The synchronicity manifests as periods of enhanced mitotic events (Fig. 1a, b) followed by mitosis-poor temporal windows that oscillate in tandem. Partial synchronicity of mitosis is a consequence of synchronised cycling that is achieved via reverse cycling [7]. In the process of reverse cycling, cells that are in G2 phase of cycle regress to G0/G1 and progress to G2 in synchrony with their neighboring cells [7]. This phenomenon is reminiscent of developmental endocycling where a reversal of cell cycle directionality from G2 to G1 phase is followed by re-entry into S phase [31]. In Drosophila, the switch that regulates the mitosis cell cycle to endocycle is triggered by activation of the Notch signaling pathway [32, 33]. Activation of the latter signaling cascade results in ectopic activation of Anaphase promoting complex and reversal of cell cycle by proteasomal degradation of its targets [33]. Given that β-catenin and Notch exhibit an antagonistic relationship [34, 35], recruitment of β-catenin to junctional complexes may trigger reversal of cell cycle via a similar mechanism. Notably, the reversal of cell cycle is markedly accelerated in neural organoids where stabilization of junctional complexes and the resultant sequestration of β-catenin lead to near-complete synchronisation of individual cells [7]. In parallel, recruitment of β-catenin into junctional complexes delays progression of cell cycle subsequent to reversal into G0/G1. This is because β-catenin *trans*-activates cyclin-D1 [21] and C-myc [22], two major drivers of cell cycle.

These findings suggest that metazoan cell cycle, unlike that of unicellular organisms, has lost its autonomy and is coupled to other cycling cells. Recruitment of β-catenin to junctional complexes [14] signals the availability of neighboring cells (i.e. the coupled state) leading to improved synchronicity of a cycling population [36]. This is because intercellular contacts stabilize cadherin-based junctions and enhance the capacity for recruitment of free cytoplasmic β-catenin. In consequence, enhanced recruitment of β-catenin to junctional complexes results in synchronisation of cycling cells at G0/G1. On the contrary, mobilization of β-catenin from junctional complexes into the free cytoplasmic pool of the protein triggers progression of cell cycle and reduces the synchronicity of cycling in the population. This mechanism is analogous to quorum sensing in bacteria [37] and triggers complete synchronisation of a cycling population beyond a specific density threshold (i.e. contact inhibition) [38]. However, unlike the binary nature of bacterial quorum sensing, metazoan quorum sensing could control the mitotic landscape in a graded manner as explained below.

## Mathematical and numerical basis of synchronisation by coupled cycling

It is noteworthy that the synchronicity of a cycling population in relation to the coupling strength (i.e. stability of adherens junctions) can be numerically predicted using the Kuramoto model. The Kuramoto model, named after the mathematician who proposed it, accurately describes the behavior of a large set of oscillators that are connected by a weak coupler [39]. In this model, intrinsic frequency of individual oscillators synchronizes to that of the others by a coupler (Fig. 1c) that accelerates the slower oscillators and decelerates the faster ones [40]. Interestingly, this model accurately predicts emergence of synchronisation in other biological systems as diverse as synchronized chirping of crickets, flashing of fireflies and schooling of fish [41]. In all these systems, activity of a weak coupler is the only requirement for emergence of self-organisation by synchronisation of a cellular population. Further, altered strength of coupling is sufficient to reprogram the temporal and spatial dimensions of a self-organising system. During cellular self-organisation, stringency of coupling could be regulated by modulating the stability of junctional complexes [7]. In addition to exogenous cues, cell-intrinsic mechanisms that alter the availability of β-catenin transcript, e.g. microRNAs, could reprogram the self-organisation dynamics [29]. Hence, combined activities of cell-extrinsic and cell-intrinsic factors determine the coupling strength and the cell cycle landscape of a cycling population. The cell cycle state of individual cells, in turn, regulates emergence of self-organisation signatures.

## Coupled cycling and programming of morphogenic landscape

**Fig. 2:**
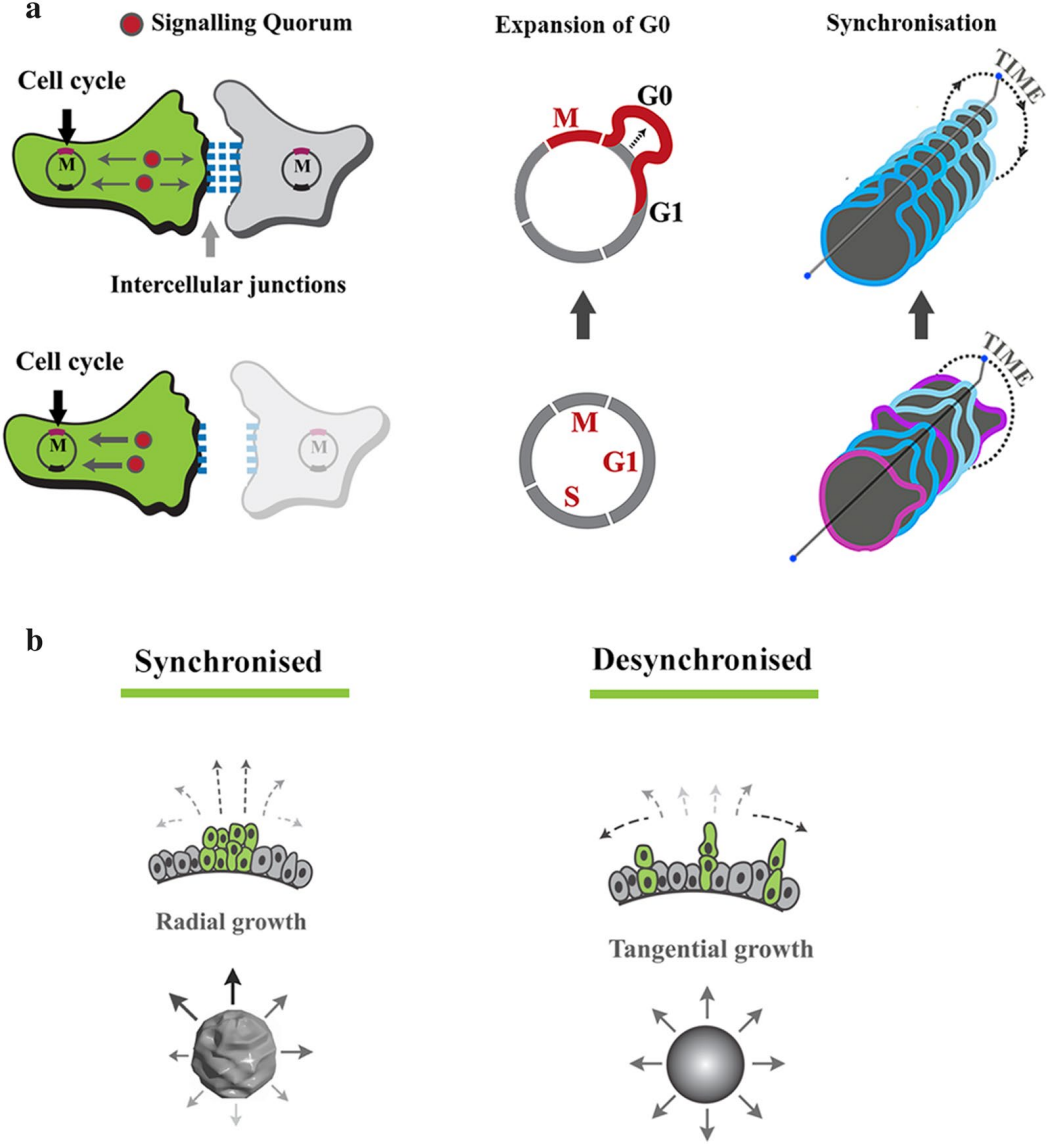
Synchronised cycling instructs multiple facets of multicellular self-organisation. **a** β-Catenin functions as a coupler of cycling cells. This is because the transcriptional activity of free cytoplasmic catenin-β1 in driving cell cycle is counterbalanced by recruitment of this protein into cadherin-based intercellular junctions. Enahnced coupling stringency (presence of neighbouring cells) leads to expansion of G0/G1 phase of cell cycle and subsequent synchronized progression into interphase at a population level. **b** Synchronised cell division leads to radial growth as opposed to tangential growth instructed by asynchronised cell division

Proliferation/differentiation dichotomy, sub-lineage differentiation outcome and spatial navigation are three major events that shape the self-organisation signature of a cellular population. Emerging evidence suggests that proliferation/differentiation dichotomy is, in part, resolved by feedback from cell cycle [42]. In fact, the activity of β-catenin, as the coupler, is sufficient to regulate cell cycle and the associated downstream phenomena. During neurogenesis, for example, constitutive signalling by stabilized β-catenin enhances the proliferative capacity of neural precursors and significant expansion of the cerebral cortex [43]. Further, β-catenin regulates the sub-lineage differentiation bias upon resolution of the proliferation/differentiation dichotomy [44]. Eventually, β-catenin interfaces with spatial cues that control the navigation of migratory cells during self-organisation [7, 45]. Hence, synchronisation by coupling or desynchronization by uncoupling robustly instructs sub-lineage differentiation outcome and spatial migration of differentiating cells due to the reprogramming of cell cycle dynamics (Fig. 2a). In the context of neurogenesis, while desynchronised cells assume a glial differentiation bias, synchronised populations (expanded G0/G1) demonstrate a neuronal differentiation bias [7]. A further corollary of synchronised mitosis is that cell division is restricted to a narrow temporal window compared to dispersion of mitotic events in an asynchronous population (Fig. 2b). Given that altered mechanical properties of mitotic cells influence the growth pattern of developing tissues [46, 47], synchronisation of division could potentially amplify this effect. For example during brain development, synchronised division of neural progenitors has the potential to alter the growth pattern from tangential to radial and to increase the gyrification index of the brain [29].

## Evolutionary perspective

Decentralised regulation of morphogenesis by self-organisation improves robustness of the developmental landscape as explained above. As such, self-organisation could also improve the heritability of various traits by canalisation [48] of development. Canalization describes the tendency for robust unfolding of a specific genotype to follow the same trajectory despite external or internal perturbations such as genomic mutations [49]. On the other hand, mutational reprogramming of the self-organisation lexicon could drive generation of novel phenotypes that are simultaneously stabilized via canalisation. Molecular evolution of the Wnt cascade clearly illustrates the latter point. Wnt morphogens activate a cascade of events that ultimately inhibit proteasomal degradation of β-catenin [50]. It can be argued that Wnt-mediated amplification of β-catenin activity mimics the uncoupled state (i.e. reduced stability of the junctional complexes) leading to an altered cellular self-organisation signature. Similarly, cell-intrinsic post-transcriptional regulation of β-catenin could have profound morphogenic consequences [29].

## Conclusion

It is proposed that coupled cycling foreshadows a fundamental adaptive change that facilitated evolution and diversification of multicellular life forms. In coupled cycling the stringency of coupling is programmed by availability and subcellular localization of β-catenin that in turn invokes specific self-organisation signatures, anatomical patterns and functional portfolios that characterize tissues and organs. It is suggested that major morphogens the function via β-catenin, such as Wnt, may alternatively be reinterpreted as modulators of coupling strength.

## Abbreviations

Ki-67: Marker of proliferation Ki-67
MCM2: Minichromosome maintenance complex component 2
C-Myc: MYC proto-oncogene
